# Mean Platelet Volume in Graves’ disease: A Sign of Hypermetabolism Rather than Autoimmunity?

**DOI:** 10.12669/pjms.334.12659

**Published:** 2017

**Authors:** Gulay Simsek Bagir, Filiz Eksi Haydardedeoglu, Okan Sefa Bakiner, Emre Bozkirli, Melek Eda Ertorer

**Affiliations:** 1 Gulay Simsek Bagir, MD. Baskent University Faculty of Medicine, Division of Endocrinology Adana, Turkey; 2 Filiz Eksi Haydardedeoglu, MD. Baskent University Faculty of Medicine, Division of Endocrinology Adana, Turkey; 3 Okan Sefa Bakiner, Associate Professor of Endocrinology, Baskent University Faculty of Medicine, Division of Endocrinology Adana, Turkey; 4 Emre Bozkirli, Associate Professor of Endocrinology, Baskent University Faculty of Medicine, Division of Endocrinology Adana, Turkey; 5 Melek Eda Ertorer, Professor of Endocrinology, Baskent University Faculty of Medicine, Division of Endocrinology Adana, Turkey

**Keywords:** Autoimmunity, Graves’, disease, Mean platelet volume

## Abstract

**Objective:**

To evaluate the impact of mean platelet volume (MPV) on predicting disease course among patients with Graves’ disease (GD).

**Methods:**

This retrospective study was performed between 2013-2016 at the Outpatient Endocrinology Clinic of Baskent University Faculty of Medicine, Adana hospital on 65 patients with GD. Among participants, 30 cases experienced thyrotoxicosis again during the first six months after discontinuing anti-thyroid drug (ATD) sessions that had been carried out for at least 12 months prior to stopping (Relapse group). We also observed 35 patients who exhibited normal thyroid functions within six months following ATD withdrawal (Remission group). MPV levels and thyroid function tests were recorded and total duration of ATD therapy was calculated for all participants.

**Results:**

The mean MPV level that was measured at the time of drug withdrawal did not differ between groups, being 8.0±1.2 fL in the Relapse group vs. 8.0±1.0 fL in the Remission group (p=0.81). However, we found that the relapse MPV was higher than the withdrawal MPV in the Relapse group (9.2±1.3 fL) than it was in the Remission group (8.0±1.2 fL, p=0.00).

**Conclusions:**

Higher relapse MPV in Relapse group but similar MPV levels in both groups at ATD withdrawal may be attributed to hypermetabolism or hyperthyroidism rather than autoimmunity of GD.

***Abbreviations:***

**BMI:** Body mass index

**GD:** Graves’ disease

**MPV:** Mean platelet volume

**TSH:** Thyroid-stimulating hormone

**TRAbs:** Thyrotropin receptor antibodies

**ATD:** Anti-thyroid drug

**fT4:** Free thyroxine

**fT3:** Free triiodothyronine

**CBC:** Complete blood count

**PTC:** Papillary thyroid carcinoma

## INTRODUCTION

Graves’ disease (GD) is an autoimmune thyroid disorder characterized by thyrotoxicosis, frequent infiltrative orbitopathy, and occasional infiltrative dermopathy.^1^-^3^ Thyrotoxicosis can cause some alterations in various body systems. In the hematopoietic system, red blood cell mass is increased due to the direct effect of thyroid hormones on the erythroid marrow and increased erythropoietin production. Platelet levels and intrinsic clotting mechanism are normal, but the concentration of factor VIII is often increased.^4^,^5^ Additionally, an increase in megakaryocytes and a decrease in platelet survival time can be observed in hyperthyroid patients.

There are three effective therapeutic options for GD: anti-thyroid drug (ATD), surgery and radioactive iodine therapy. Since there is no definitive conclusion as to which option is most effective, physician and patient preferences often dictate the choice. Anti-thyroid drugs may alter the course of autoimmune process, but the optimal duration of treatment using them has not been clearly determined. A long term medical therapy lasting at least 12 months is usually administered and then withdrawn if serum thyroid stimulating hormone (TSH) levels return to normal.^6^ The average rate of remission is between 30% and 50% after ATD withdrawal.^2^,^7^

The course of GD is variable. In some patients, thyrotoxicosis persists, while it exhibits remission after ATD withdrawal in others. About 75% of relapses occur during the first three months after drug withdrawal, with most of the remainder occurring during the subsequent 6 months.^8^

There are some factors that may predict the outcome of GD after drug withdrawal. For example, the severity of hyperthyroidism at initial diagnosis is associated with higher relapse rates. Levels of thyrotropin receptor antibodies (TRAbs) are decreased by medical treatment but may predict recurrence if they persist during this regimen. Additionally, remission rates are reported to be lower in men, younger patients, smokers and patients with larger goiter size and ophthalmopathy upon diagnosis.^9^-^12^

Mean platelet volume (MPV) is a machine-calculated measurement of the average size of platelets that can be used to determine platelet production and destruction rate in bone marrow. Inflammation acts an important stimulant for platelets; consequently, large platelets that release a variety of pro-inflammatory and thrombotic factors are more active. We can therefore consider MPV to be a simple sign of subclinical inflammation, and it also has been reported to be a marker of inflammatory disease activity.^13^-^14^

In this study, we aimed to investigate the relationship between MPV and disease course in patients with GD. We also inquired into the association between treatment outcome and clinical and other laboratory variables. After careful consideration of these factors, we proposed that MPV measured at ATD withdrawal will be higher in relapse GD.

## METHODS

This retrospective study was performed at the Outpatient Endocrinology Clinic of Baskent University Faculty of Medicine, Adana hospital between the years 2013 and 2016. The medical records of GD patients who were followed up at least six months after discontinuing a minimum one year course of ATD treatment were recruited to the study. All participants were euthyroid at ATD withdrawal.

The diagnosis of GD was based on standard clinical and laboratory criteria, including clinical symptoms of hyperthyroidism, thyroid ultrasound findings, increased concentrations of free thyroxine (fT4), free triiodothyronine (fT3) and suppressed TSH concentrations in all patients. Additionally, measurement of TRAbs, determination of high radioactive iodine uptake and/or diffuse uptake at thyroid scintigraphy were performed in some patients.^6^ All ATD treatment data was recorded from medical documents.

Our study excluded Graves’ disease patients with known histories of the following conditions: cirrhosis, chronic kidney disease, cancer, cardiovascular disease, diabetes mellitus, and severe obesity (body mass index (BMI)>35kg/m^2^), infections, chronic inflammatory diseases that include collagen tissue disease and inflammatory bowel disease, haematological diseases that include haemoglobinopathy, and blood coagulation disorders. Furthermore, patients undergoing treatment with diuretics, antihyperlipidemic agents, anticoagulants, and corticosteroids were excluded. Smokers were also excluded.

All patients were treated with propylthiouracil or methimazole by a dose titration regimen. According to disease course, patients were divided into two groups, the Relapse and Remission groups. Relapse group patients experienced thyrotoxicosis again during the first six months after discontinuation of at least 12 months of ATD sessions. The Remission group consisted of GD patients who exhibited normal thyroid functions within six months following ATD withdrawal, as mentioned above.

Serum TSH, fT4, fT3 and MPV levels were recorded at the time of ATD withdrawal and relapse in the Relapse group and at the time of drug withdrawal in the Remission group. The total duration of ATD therapies was calculated, as well. The study was approved by the Local Ethics Committee of Baskent University, (Project no: KA 15/385).

### Laboratory Analysis

In order to measure each participant’s complete blood count (CBC), a venous blood sample was obtained and collected in K- ethylenediaminetetraacetic acid (EDTA) tubes and measured by Siemens Advia 2120i Haematology System (Siemens Healthcare Inc. Tarrytown, NY USA). The MPV measurements were performed immediately in our laboratory with a reference value for MPV of 7.2-11.1 fL. Automated chemiluminescent immunoassays performed by the Advia Centaur XP Immunoassay system (Siemens Healthcare İnc. Tarrytown, NY USA) were used to measure fT4 (normal range:11.5-22.7 pmol/L), fT3 (normal range:3.5-6.5 pmol/L) and TSH (normal range:0.4-4.6 uIU/ml).

### Statistical Methods

Statistical analysis was performed using the statistical package *SPSS software* (Version 17.0, SPSS Inc., and Chicago, IL, USA). If continuous variables were normal, they were described as the mean±standard deviation (p>0.05 in Kolmogorov-Smirnov test or Shapira-Wilk (n<30)); however, if the continuous variables were not normal, they were described as the median. Groups of normally distributed data were compared using Student T test or One Way ANOVA for normally distributed data, while Mann Whitney U test or Kruscall Wallis tests were used for groups of data not normally distributed. The categorical variables between the groups were analysed using the Chi square test or Fisher Exc. test. Pre-post measures data were analysed using the Paired T test or Wilcoxson test. Values of p<0.05 were considered statistically.

## RESULTS

Sixty-five cases were recruited from 245 GD patients depending on the excluding criteria previously mentioned. The Relapse group contained 30 patients, while the Remission group was made up of 35 patients. The general characteristics of the patients are shown in Table-1. Significant differences among the groups did not exist in terms of age, gender or body mass index (BMI). The mean duration of total ATD therapy was calculated as 22±7.8 months in the Relapse group and 16.6±3.1 months in the Remission group. Our findings showed that the therapy duration was significantly shorter in remission group (p=0.00).

**Table-I:** General characteristics of Graves patients.

	*Relapse group (n=30)*	*Remission group (n=35)*	*P*
Age (years)	44.3 ± 14.9	43.6 ± 14.9	0.83
Gender, F (%)	23 (76.6)	26 (74.2)	0.82
BMI (kg/m^2^)	25.9 ± 4.7	26.8 ± 4.7	0.44
Duration of ATD therapy (months)	22. ± 7.8	16.6 ± 3.1	0.00

***Note:*** ATD- antit-hyroid drug, Values are mean ± SD

According to our findings, the mean MPV level measured at the time of drug withdrawal did not differ between groups, being 8.0±1.2 fL in the Relapse group and 8.0±1.0 fL in the Remission group (p=0.81). The mean TSH level that was measured at the time of drug withdrawal was higher in the Remission group at 2.0±1.0, as opposed to 1.5±1.0 uIU/ml in the Relapse group (p=0.03, Table-II). We found that the relapse MPV was higher than the withdrawal MPV in the Relapse group at 9.2±1.3 fL vs 8.0±1.2 fL, p=0.00, respectively. Even though the relapse group had higher relapse MPV values, most of them were within the normal limits. Only two (6.6%) were above limits. Additionally, the Relapse group had a statistically higher relapse MPV than the withdrawal MPV of the Remission group at 9.2±1.3 vs 8.0±1.0 (p=0.00, Table-III).

**Table-II:** Comparision of laboratory values of groups at ATD withdrawal.

	*Relapse group (n=30)*	*Remission group (n=35)*	*P*
Withdrawal MPV (fL)	8.0±1.2	8.0±1.0	0.81
Withdrawal TSH (uIU/ml)	1.5±1.0	2.0±1.0	0.03
Withdrawal fT3 (pmol/L)	4.8±1.2	4.7±0.5	0.46
Withdrawal fT4 (pmol/L)	14.8±2.9	13.6±1.6	0.04

***Note:*** ATD- anti-thyroid drug, MPV- mean platelet volume, Values are the mean ± SD.

**Table-III:** Comparison of laboratory values of groups according to disease outcome.

	*At Relapse in Relapse group (n=30)*	*At withdrawal in Remission group (n=35)*	*P*
MPV(fL)	9.2±1.3	8.0±1.0	0.00
TSH (uIU/ml)	0.05±0.08	2.0±1.0	0.00
fT3 (pmol/L)	9.7 (3.7-30)	4.7±0.5	0.00
fT4 (pmol/L)	26.3 (14-65)	13.6±1.6	0.00

***Note:*** MPV- mean platelet volume, Values are the mean ± SD or median (minimum–maximum)

## DISCUSSION

Mean platelet volume (MPV) is an automated measurement of average size of platelets in whole blood that adds no extra cost or effort to a full blood count. The positive relationship between MPV and inflammation has been shown many times before.^13^,^14^ Platelets with higher volume are considered to be involved in inflammation; thus, MPV levels have been reported to be a useful biomarker of subclinical inflammation in patients with inflammatory diseases like Crohn’s disease and rheumatoid arthritis.^13^,^15^ The MPV levels are also known to be affected by thyroid hormone status. In the present study, we investigated the MPV levels of GD patients who relapsed and stayed in remission during at least six months of follow-up, after a minimum 12-month course of ATD therapy that was withdrawn when the cases were euthyroid. The mean MPV of the groups was indifferent at drug withdrawal but was significantly higher in the Relapse group at relapse time.

Current medical literature covers several trials reporting MPV levels in different types of thyroid diseases. Studies investigating the relationship between thyroid functions and MPV levels demonstrate conflicting results. For example, Lippi and colleagues demonstrated a significant positive correlation between MPV and serum TSH values, whereas Ren et al. could not confirm the relationship.^16^,^17^

In another study, Baldane and colleagues found a significant decrease in MPV levels of papillary thyroid carcinoma (PTC) patients after surgical treatment, and they also noted that preoperative MPV levels of PTC cases were significantly higher than the ones with benign goiter. They suggest that MPV may be used as a biomarker in the diagnosis of PTC and may point to the role of platelets in malignant diseases with inflammatory backgrounds.^18^

Due to unpredictable responses, it is difficult for physicians to choose the optimal treatment regimen in GD. In Europe, Latin America and Japan, ATDs are considered the first choice of therapy.^19^ Determining predictors of relapse following ATD treatment can undoubtedly improve patient management. We must consider the fact that several factors have been shown to indicate poor prognosis at the onset of the disease, including a large goiter size, severe thyroid dysfunction and high levels of TRAbs.^12^,^20^ There are also aberrant expression and secretion of several cytokines thought to be the predictors of prognosis. Serum levels of cytokines, such as IL-4, IL-6 and IL-10, are found to be higher in patients with refractory GD when compared to those in remission.^21^ However, the high cost of determining these parameters limits their use. Responding to the obvious need for cost effective and widely available tools that may predict disease course, we theorized that MPV would fill that void in the medical community.

The higher MPV values in our Relapse group may be explained in several ways. The first possible explanation comes from a haematological point of view and is based on the shortened platelet survival that is observed in patients with hyperthyroidism. It is very well known that platelet size decreases with their age. As patients with hyperthyroidism have greater numbers of younger platelets, MPV levels will inevitably be higher than that of the euthyroid ones.^22^ Accordingly, a study comparing the MPV levels before and after ATD therapy has found a significant decrease after three weeks of ATD therapy following euthyroidism. This study has clearly shown that platelet lifespan is significantly shortened in patients with hyperthyroidism, and higher MPV levels are therefore proposed to be metabolically, not immunologically, mediated phenomena.^23^ Our second possible explanation comes from an immunological point of view. Higher MPV levels that were measured at relapse time in the Relapse group may be a marker of inflammatory disease activity. This conclusion is naturally drawn from the fact that the Relapse group has active GD. Our first explanation appears to be more valid, since the cases in both groups exhibited similar MPV levels when they were euthyroid at ATD withdrawal time. The higher MPV level at relapse points to the shortened platelet lifespan with hyperthyroidism.

Our study applied strict excluding criteria in order to eliminate any factors that may interfere with MPV. While this strategy surely strengthened the present study, it inevitably resulted in a relatively low number of participants. We hope this fact will be appreciated by those considering our findings.

### Limitations of the study

Its retrospective design is one of them. The relatively short follow-up time after ATD withdrawal may be considered to be another limitation. However, knowing that about 75% of relapses occur during the first three months after drug withdrawal, we believe that our approach is reasonable.

## CONCLUSION

In this study, we found higher relapse MPV in the Relapse group but discovered similar MPV levels in both groups at the point of ATD withdrawal. This finding may be attributed to hypermetabolism of hyperthyroidism rather than autoimmunity of GD. Further studies are needed to confirm our results.

### Authors’ Contribution

**GSB:** Conceived, data collection, designed, writing manuscript.

**FEH:** Designed, data collection. **OSB:** Conceived, data collection.

**EB:** Designed, data collection. **MEE:** Did statistical analysis, conceived and editing.
